# Opportunistic diseases in marine eukaryotes: Could Bacteroidota be the next threat to ocean life?

**DOI:** 10.1111/1462-2920.16094

**Published:** 2022-08-04

**Authors:** Jennifer Hudson, Suhelen Egan

**Affiliations:** ^1^ Centre for Marine Science and Innovation, School of Biological, Earth and Environmental Sciences The University of New South Wales Sydney Australia

## Abstract

Bacteria within the phylum Bacteroidota (Bacteroidetes) are known to cause devastating and widespread disease outbreaks in marine eukaryotic hosts. However, with few pathogens described in detail, their prevalence and virulence strategies remain largely unknown. Here, we systematically reviewed the literature to evaluate the current understanding of Bacteroidota that cause disease in marine hosts. Isolates affiliated with the genera *Tenacibaculum* and *Aquimarina* (Flavobacteriaceae) were the most widely reported and characterized pathogens. Although cultured isolates were predominantly Flavobacteriia, culture‐independent studies also found classes Bacteroidia, Cytophagia and Sphingobacteriia associated with disease. We found that pathogenic marine Bacteroidota largely conformed to an opportunistic lifestyle but could also act as secondary pathogens or were involved in polymicrobial diseases. Many diseases were also associated with an environmental stressor, especially those affecting coral, macroalgae and fish. Key virulence traits included the production of adhesins and host tissue‐degrading enzymes. Overall, the nature of disease involving Bacteroidota pathogens appears to be an outcome of complex host–pathogen–environment interactions; however, our understanding of virulence remains limited by the lack of functional characterization studies. This is concerning as Bacteroidota have the potential to emerge as a serious threat to marine ecosystems and aquaculture industries, driven by global changes in ocean conditions.

## INTRODUCTION

Bacteria belonging to the phylum Bacteroidota are broadly distributed across a diverse range of ecological niches on earth, including host‐associated microbiomes, soil, polar, freshwater and marine habitats (Thomas et al., 2011). Originally referred to as the *Cytophaga‐Flavobacterium‐Bacteroides* group, this phylum has been the subject of extensive taxonomic revision in recent years (García‐López et al., 2019; Hahnke et al., 2016), including a recent revision of the phylum name from Bacteroidetes to Bacteroidota (Oren & Garrity, 2021). Since the phylum was first described by Krieg et al. (2010), the number of species has more than doubled, and is now comprised of six classes with Flavobacteriia, Cytophagia and Bacteroidia having the most described species (García‐López et al., 2019, Hahnke et al., 2016). Bacteroidota have traditionally been classified as chemoheterotrophic Gram‐negative bacteria that are non‐motile (except by gliding), non‐spore forming and can be either aerobic or anaerobic (Krieg et al., 2010). Although genomic approaches have improved our understanding of their core functions and phylogeny, the diversity and complexity of this phylum mean that it remains relatively uncharacterised (García‐López et al., 2019; Hahnke et al., 2016; Munoz et al., 2016; Munoz et al., 2020).

In the oceans, Bacteroidota constitute a high proportion of the bacterial biomass and are largely affiliated with the order Flavobacteriales (El‐Swais et al., 2015; Glöckner et al., 1999; Kirchman et al., 2010; Wietz et al., 2010; Yilmaz et al., 2016; Zhang et al., 2019a). Marine Bacteroidota are most widely recognized for their important role in biogeochemical cycling due to the diverse array of carbohydrate‐active enzymes (CAZymes) and peptidases typically encoded within their genomes (Fernández‐Gomez et al., 2013; Gavriilidou et al., 2020). This attribute provides Bacteroidota with the ability to degrade an assortment of complex plant‐ and animal‐derived polymers (such as agar, cellulose and chitin) and hence they are generally considered to be primary degraders of organic matter (Fernández‐Gomez et al., 2013; Thomas et al., 2011). Bacteroidota also employ several unique strategies to further enhance the efficiency of their carbohydrate metabolism including the phylum‐specific type 9 secretion system (T9SS). The T9SS is highly effective in the secretion of CAZymes and other extracellular proteins while additionally mediating surface‐associated gliding motility (Lasica et al., 2017; McBride, 2019). Together, these traits ultimately confer a key competitive advantage allowing Bacteroidota members to successfully colonize and thrive in diverse ecological niches (Munoz et al., 2020).

As a result of their versatile metabolic profile, Bacteroidota are often found in association with nutrient‐rich eukaryotic organisms where they have evolved complex symbiotic relationships. In some instances, Bacteroidota encompass mutualistic or commensal functions such as promoting larval settlement and development in sea sponges (Li et al., 2021; Webster et al., 2013) and correct morphogenesis of green algae (Marshall et al., 2006; Matsuo et al., 2003; Nakanishi et al., 1996). Bacteroidota are also consistently identified in the gut microbiomes of omnivorous fishes and mammals where they may facilitate the breakdown of ingested polysaccharides (Nelson et al., 2015; Smriga et al., 2010; Sullam et al., 2012).

Conversely, there are reports of Bacteroidota that have adopted a pathogenic lifestyle, leading to severe negative health outcomes for the host. Arguably, the most widely documented marine pathogens within Bacteroidota belong to the genus *Tenacibaculum* within the family Flavobacteriaceae (Nowlan et al., 2020). Multiple species of *Tenacibaculum* have been implicated as aetiological agents of tenacibaculosis disease which affects a range of commercially valuable fish species globally (Avendaño‐Herrera et al., 2006; Fernández‐Álvarez & Santos, 2018; Nowlan et al., 2020). Although considerably less is known about other pathogenic marine Bacteroidota, the advent of culture‐independent techniques such as 16S rRNA gene amplicon sequencing has provided evidence for the involvement of a range of Bacteroidota across numerous marine diseases. For example, studies in corals found that Flavobacteriaceae are abundant in individuals affected by white band disease and reduced in healthy hosts (Gignoux‐Wolfsohn & Vollmer, 2015). Similar patterns have also been seen in diseased sponges (Webster et al., 2008), sea stars (Lloyd & Pespeni, 2018), macroalgae (Zozaya‐Valdes et al., 2015) and lobsters (Meres et al., 2012; Ooi et al., 2020). An increasing number of pathogenic species are also being isolated, characterized and implicated as aetiological agents of disease. Moreover, some of these pathogens have been hypothesised to be opportunistic in nature due to their presence on healthy tissue.

Overall, the body of research concerning the prevalence, virulence strategies and pathogenicity of marine Bacteroidota remains remarkably limited, especially when compared to the scope of research on other marine pathogens. Hence, this study aims to review the available literature to: (i) determine the prevalence and range of bacterial pathogens belonging to Bacteroidota that cause disease in marine eukaryotic hosts, (ii) examine their virulence strategies and external factors influencing host–pathogen dynamics and (iii) identify limitations in our current understanding and avenues for future research.

## LITERATURE SEARCHING AND FILTERING

Peer‐reviewed primary literature was retrieved using PubMed, Scopus and Web of Science on 10 May 2022. The following search string was applied: [(Bacteroid* OR Chitinophag* OR Cytophag* OR Flavobacter* OR Saprospir* OR Sphingobacter*) AND (marine OR ocean OR seawater) AND (disease* OR pathogen* OR virulen*)], with Scopus records limited to title/abstract/keywords.

The literature search yielded 590 (PubMed), 446 (Scopus) and 955 (Web of Science) publications. Search results were exported to EndNote citation management program and duplicates were removed resulting in 1258 unique records. Unique publications were manually screened for inclusion. A total of 46 additional studies identified during the screening process (i.e. via citations within articles) were also included.

Studies were included if they:

- involved the isolation of a novel Bacteroidota species or strain that was directly associated with a disease phenotype or mortality outcome in a marine eukaryotic host,
- used culture‐independent methods to identify a positive association of Bacteroidota with a disease phenotype in a marine host,
- characterized virulence traits of known marine Bacteroidota pathogens,
- were published in English.

Studies were excluded if they:

- were unrelated to marine systems or hosts
- concerned the host response to infection,
- solely focused on disease management or
- were not directly linked to a disease phenotype or mortality outcome.

Studies related to *Flavobacterium psychrophilum* infections in diadromous fish (including salmonids and eel) were not included in this review as these studies primarily concerned freshwater systems and have been reviewed elsewhere (Loch & Faisal, 2015; Starliper, 2011; Wahli & Madsen, 2018). Other Bacteroidota pathogens affecting diadromous fishes were included if the studies were specifically performed within a marine context.

## PREVALENCE AND HOST RANGE OF PATHOGENIC MARINE BACTEROIDOTA

**FIGURE 1:**
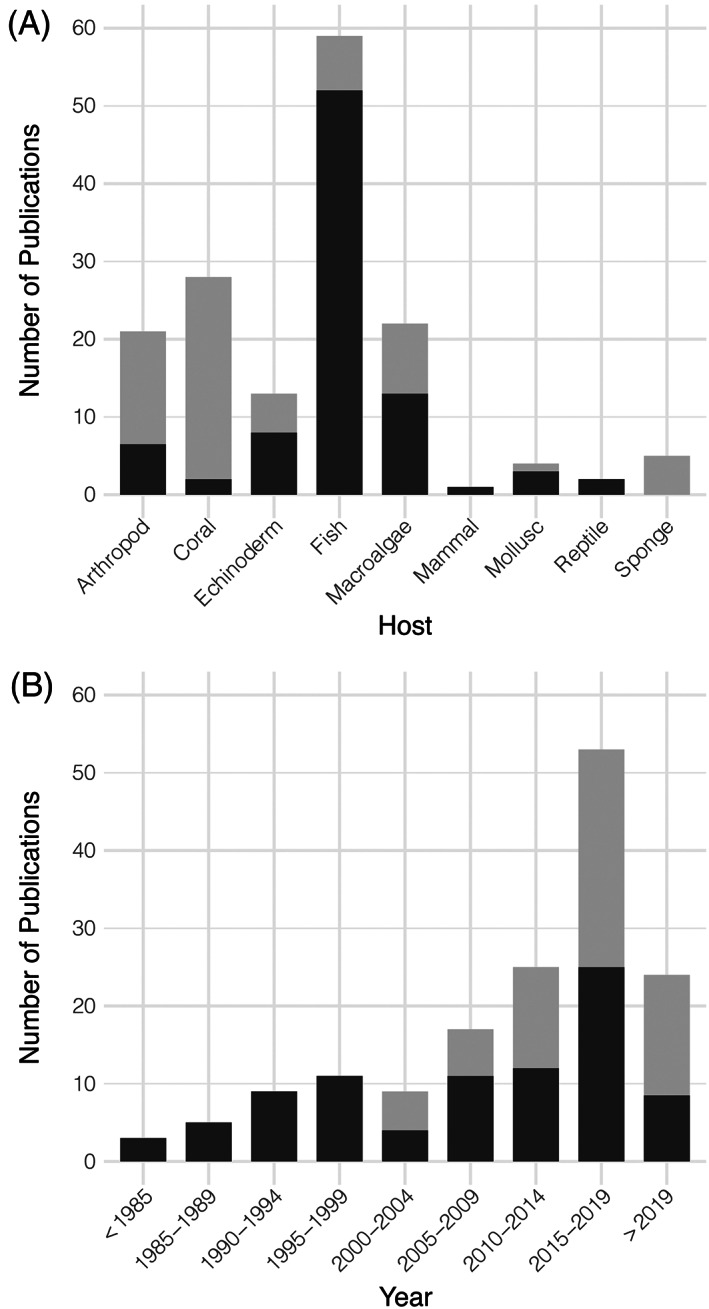
(A) Number of studies related to Bacteroidota in disease by host type. (B) Number of reports related to pathogenic Bacteroidota over time. Dark bars represent the number of culture‐dependent studies and light bars represent the number of culture‐independent studies

Members of the phylum Bacteroidota have been implicated as the causal agent of disease across a range of marine eukaryotic hosts [Figure 1(A)]. In this study, we identified 155 reports of disease where Bacteroidota were positively involved, with 43% of these studies utilizing culture‐independent techniques and 56% applying culture‐dependent methods, and one study using both approaches (Table S1).

Putative pathogenic Bacteroidota have been cultured from a range of marine eukaryotic hosts, primarily from fish and macroalgae, but also from arthropods, corals, echinoderms, mammals, molluscs and reptiles [Figure 1(A) and Table S2]. Here, most of these putative pathogens belong to the family Flavobacteriaceae. Few isolates were also assigned to Cytophagaceae; however, these classifications were derived from biochemical traits prior to the taxonomic revision of Bacteroidota and have not been verified (Dungan et al., 1989; Largo et al., 1995a). Pathogens belonging to the genus *Tenacibaculum* were the most well‐characterized, which is not unexpected due to the severe nature of tenacibaculosis disease that results in high mortality rates in fish with substantial economic losses (Avendaño‐Herrera et al., 2006). The genus *Aquimarina* was also represented in the literature with numerous studies covering infection dynamics, phenotypic characterization and genome analysis across pathogens of crustacean and macroalgal hosts (Table 1). No specific reports concerning the virulence strategies of other pathogenic genera including *Lacinutrix*, *Kordia*, *Chryseobacterium* and *Gaetbulibacter* were identified (Table 1). Most of these pathogens were only recently identified and so more research is needed to characterize the mechanisms they use to cause damage to marine plants and animals.

**TABLE 1:** List of pathogenic marine Bacteroidota isolates where pathogenicity has been confirmed using an infection experiment fulfilling, or partially fulfilling, Koch's postulates

Pathogen name^a^	Host organism(s)	Disease name/phenotype	Reference
*Aquimarina hainanensis*	Giant mud crab, Gazami crab, Brine shrimp	Tissue necrosis, mortality events	Dan and Hamasaki (2015), Midorikawa et al. (2020)
*Aquimarina latercula*	*Gracilaria lemaneformis* (Red algae)	Mid‐thallus bleaching	Liu et al. (2019)
*Aquimarina* sp. AD1	*Delisea pulchra* (Red algae)	Mid‐thallus bleaching	Kumar et al. (2016), Hudson et al. (2019)
*Aquimarina* sp. BL5	*Delisea pulchra* (Red algae)	Mid‐thallus bleaching	Kumar et al. (2016), Hudson et al. (2019)
*Aquimarina* sp. I32.4	American lobster	Epizootic, enzootic and impoundment shell disease	Chistoserdov et al. (2005), Chistoserdov et al. (2012), Quinn et al. (2012)
*Aquimarina* sp. TRL1	Ornate spiny lobster, Slipper lobster	White leg disease	Ooi et al. (2020)
*Chryseobacterium scophthalmum*	Turbot	Gill hyperplasia, haemorrhagic septicaemia	Mudarris and Austin (1989), Mudarris et al. (1994)
*Cytophaga* sp. P25	*Kappaphycus alvarenzii*, *Eucheuma denticulatum* (Red algae)	Ice–ice whitening	Largo et al. (1995a), Largo et al. (1995b)
Cytophaga/Flavobacterium like	*Chondrus crispus* (Red algae)	Green spot/rot disease	Craigie and Correa (1996)
*Flavobacterium columnare* FK401	Ring plate coral	Black band disease	Rahmi et al. (2020)
*Flavobacterium* sp.	*Gracilaria verrucosa* (Red algae)	Ice–ice whitening	Zainuddin et al. (2019)
*Flavobacterium* sp. LAD‐1	*Porphyra yezoensis* (Red algae)	Anaaki disease	Sunairi et al. (1995)
*Flavobacterium‐Cytophaga* group bacteria	*Gracilaria conferta* (Red algae)	Apical necrosis	Weinberger et al. (1997)
*Gaetbulibacter saemankumensis*	*Porphyra yezoensis* (Red algae)	Suminori disease	Mine et al. (2009)
*Ichthyobacterium seriolicida*	Japanese amberjack	Bacterial haemolytic jaundice	Takano et al. (2016), Matsuyama et al. (2018)
*Kordia algicida*	*Agarophyton vermiculophyllum* (Red algae)	Tip bleaching	Saha and Weinberger (2019)
*Lacinutrix venerupis*	European sea bass, Gilt‐head bream	Fin erosion, haemorrhaging, mortality	López et al. (2017)
*Tenacibaculum dicentrarchi*	Atlantic salmon	Tail rot, frayed fins, gill damage, mortality events	Avendaño‐Herrera et al. (2016), Klakegg et al. (2019)
*Tenacibaculum disolor*	Senegalese sole	Tenacibaculosis	Piñeiro‐Vidal et al. (2007), Piñeiro‐Vidal et al. (2008)
*Tenacibaculum finnmarkense*	Atlantic salmon	Tenacibaculosis	Småge et al. (2016), Småge et al. (2018)
*Tenacibaculum maritimum*	Multiple fish hosts	Tenacibaculosis	Wakabayashi et al. (1986), Avendaño‐Herrera et al. (2006), Nowlan et al. (2020)
*Tenacibaculum maritimum‐*like	Whiteleg shrimp	Lesions, mortality events	Mouriño et al. (2008), Avendaño‐Herrera (2009)
*Tenacibaculum ovalyticus*	Atlantic halibut	Larval mortality	Bergh et al. (1992), Hansen et al. (1992), Skiftesvik and Bergh (1993)
*Tenacibaculum piscium*	Atlantic salmon	Winter ulcers	Olsen et al. (2011), Olsen et al. (2020)
*Tenacibaculum soleae*	Wedge sole, Senegalese sole, Brill, European sea bass	Tenacibaculosis	López et al. (2010) Castro et al. (2014)
*Tenacibaculum soleae*	Pacific oyster	Lesions	Burioli et al. (2018)
*Tenacibaculum* sp. F‐2	Sea urchin	Spotting disease	Tajima et al. (1997a), Tajima et al. (1997b), Taniguchi et al. (2006)
*Tenacibaculum* sp. Pbs‐1	Akoya pearl oyster	Black spot shell disease	Sakatoku et al. (2018)

^a^ Pathogen name: taxonomically validated names, isolate name or isolate best BLAST match as reported by authors.

In cases where putative Bacteroidota pathogens were cultured, only 27 isolates had their pathogenicity confirmed using infection experiments (Table 1). This number does not include studies in *T*. *maritimum* for which pathogenicity has been demonstrated in a range of fish species. Many reports identified here described the isolation of bacteria from diseased hosts and subsequent biochemical characterization was only pursued for taxonomic purposes. Therefore, further investigation is needed to understand their ecological importance in relation to disease in eukaryotic hosts.

**FIGURE 2:**
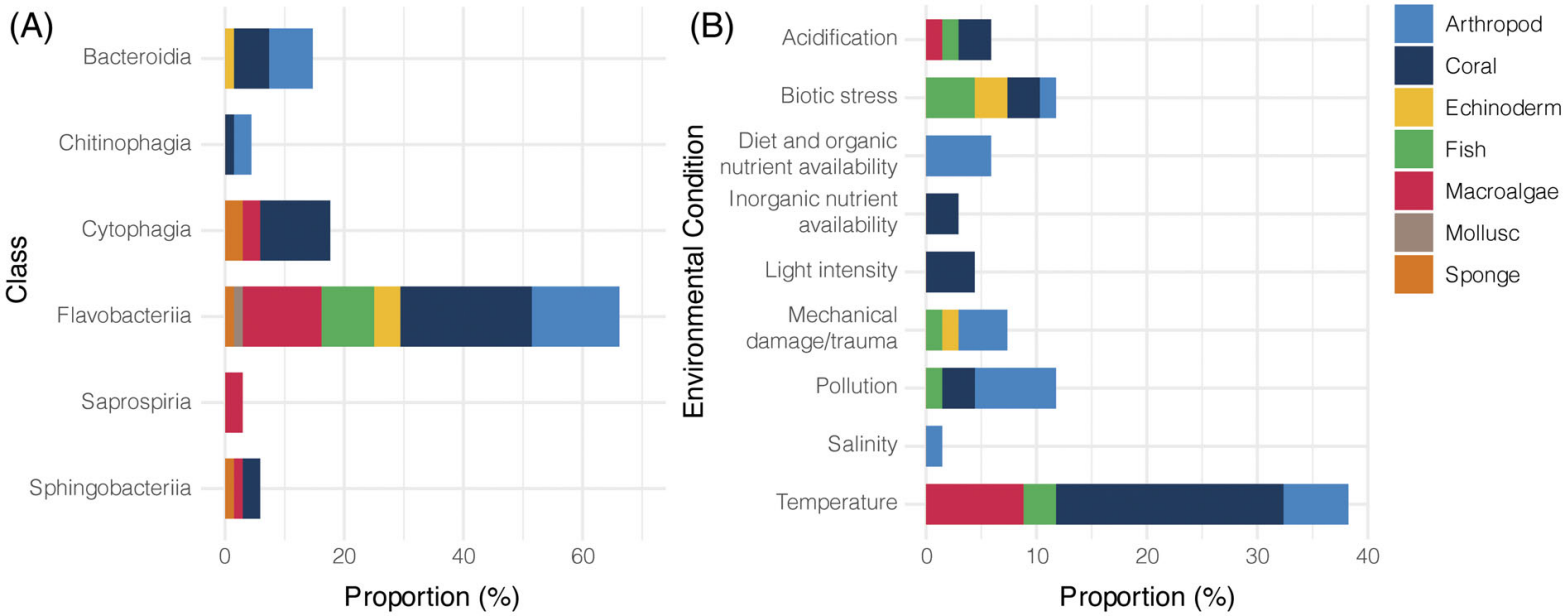
(A) Number of times each class level taxa within the phylum Bacteroidota was reported in a disease phenotype using culture‐independent methods, as a proportion of total culture‐independent studies. (B) Number of times a culture‐independent study reported an environmental condition associated with a disease phenotype, as a proportion of total culture‐independent reports. In both graphs, bars are coloured to provide a breakdown of the host from which a particular taxon was identified (A), or the host in which the environmental condition was associated with (B). Note: Bars do not add up to 100% due to multiple taxa or environmental conditions being reported

A large number of studies published over the last decade utilized culture‐independent methods to implicate Bacteroidota in disease [Figure 1(B)]. A high proportion of these studies applied 16S rRNA gene sequencing techniques, including DGGE, cloning and next‐generation sequencing technologies, with only seven studies characterizing putative gene functions using meta‐genomic, meta‐transcriptomic and meta‐proteomic techniques. Culture‐independent studies generally identified a broader taxonomic range of Bacteroidota, including Bacteroidia, Sphingobacteriia and Cytophagia, in addition to Flavobacteriia, also represented in disease phenotypes [Figure 2(A)]. Although limited information is available surrounding pathogenic marine members of Bacteroidia, Sphingobacteriia and Cytophagia, these findings suggest that they may play a yet unknown role in marine diseases. Studies that employed culture‐independent methodologies also examined a greater number of ecologically relevant host species, particularly corals and sponges, in contrast with culture‐dependent studies which had a greater focus towards species farmed in aquaculture systems [Figure 1(A)].

## HOST–PATHOGEN DYNAMICS

We found most marine Bacteroidota that were linked to disease could be classified as opportunistic pathogens as they were also associated with healthy hosts and typically only caused disease symptoms following an environmental perturbation [Figure 2(B) and Table S2]. For example, *Tenacibaculum maritimum* was categorized as an opportunistic pathogen where the onset of disease is associated with increased temperature and salinity, and reduced water quality (Avendaño‐Herrera et al., 2006). Many other putative pathogens, especially those identified by culture‐independent methods, were not explicitly identified as opportunistic but were found to normally reside on the host surface and were enriched on diseased tissue.

Overall, diseases reported in corals and macroalgae were most frequently linked to an environmental stressor, such as changes in salinity, light, aeration and nutrient availability, with temperature being the most reported stress [Figure 2(B) and Table S2]. In macroalgal hosts, diseases caused by Bacteroidota appear to predominantly affect Rhodophyta (red algae) with fewer cases of disease reported in brown and green algae (Table 1). This includes ice–ice whitening in *Gracilaria verrucosa* and *Kappaphycus alvarezii*, and bleaching diseases in *Delisea pulchra*, *Gracilaria lemaneformis* and *Agarophyton vermiculophyllum*. In many cases of macroalgal disease, natural disease outbreaks typically occur in warmer seawater temperatures (Largo et al., 1995b; Zainuddin et al., 2019). Thermal stress is broadly considered to have negative effects on macroalgal physiology by eliciting damage to cellular structures which in turn leads to a downregulation in non‐essential metabolic processes (Xu et al., 2014) including reduced chemical defences (Campbell et al., 2011). Reduced host immune functioning may present an opportunity for commensal Bacteroidota to take advantage and exploit the polysaccharide‐rich resources of the host resulting in tissue damage. Most coral diseases identified here were similarly associated with increased seawater temperatures, suggesting that commensal Bacteroidota may function advantageously under these environmental conditions.

In other cases, such as shell diseases in lobsters, black spot shell disease in the Akoya pearl oyster, and Suminori disease in *Porphyra yezoensis*, the onset of disease was aided by pre‐existing surface damage (Table S2). For example, infection experiments in epizootic shell disease of lobsters showed that disease caused by the chitinolytic pathogen *Aquimarina* sp. I32.4 only occurred when the dermal layer was compromised to expose the underlying chitin layer (Quinn et al., 2012). Damage originating from microeukaryotes could also contribute to disease outcomes involving Bacteroidota, such as the gastropod grazing of a sea urchin (Becker et al., 2007) and amoeba and lice infections in salmon (Bowman & Nowak, 2004; Llewellyn et al., 2017; Slinger et al., 2020). Trauma to the external layer of eukaryotic hosts, either from biotic or abiotic means, can compromise the innate defences of the host, providing a route of entry for opportunistic bacteria to proliferate. This is a concern for commercially reared eukaryotes where intensive farming practices, such as high stocking density and handling processes, can result in trauma and provide favourable conditions for parasites to thrive (Bowman & Nowak, 2004; Chistoserdov et al., 2012; Llewellyn et al., 2017; Ogawa & Yokoyama, 1998; Smolowitz et al., 2014).

We also identified the involvement of Bacteroidota in polymicrobial diseases. Black band disease in coral is a well‐established model for polymicrobial disease in marine systems, consisting of a complex microbial consortium of cyanobacteria, heterotrophic and sulfur‐cycling bacteria (Frias‐Lopez et al., 2004). Of the publications employing culture‐independent methods identified in this study, 10 identified the positive involvement of Bacteroidota in coral black band disease. One study additionally observed black band disease on coral following inoculation with a strain identified as *Flavobacterium columnare* FK401 (Rahmi et al., 2020). Epizootic shell disease in lobster is also thought to be polymicrobial, where *Aquimarina* sp. I32.4 colonization is followed by a succession of other microorganisms collectively contributing to the disease phenotype (Chistoserdov et al., 2012; Meres, 2016; Meres et al., 2012; Quinn et al., 2012).

It is also possible that many Bacteroidota were isolated from diseased tissue because they act as secondary pathogens or saprophytic colonizers which proliferate on decaying tissue to exploit the available nutrients. Although no studies reviewed here specifically identified Bacteroidota as secondary pathogens, several studies did detect the involvement of Bacteroidota in diseases with a known aetiological agent. For example, the aetiology of ‘Syndrome 93’ in white leg shrimp (Costa et al., 1998; Saulnier et al., 2000), skin ulceration disease in sea cucumber (Zhang et al., 2019b) and white spot disease in crustaceans (Ding et al., 2017) were attributed to an infection with *Vibrio penaeicida*, *Vibrio splendidus* and white spot syndrome virus, respectively. Yet, in each case, Bacteroidota were found to be enriched on the diseased tissue. As specialist degraders of organic matter, Bacteroidota may have an affinity for decaying tissue where they can utilize complex polysaccharides that other bacteria are not capable of metabolizing (Larsbrink & Mckee, 2020; Thomas et al., 2011). However, even in situations where Bacteroidota do not directly initiate disease, they can play a synergistic role in the progression of disease by exacerbating damage and escalating mortality rates. This concern is becoming increasingly apparent in freshwater aquaculture, where secondary infections with *Flavobacterium* spp. led to higher mortality rates in fish affected by viral primary pathogens (Adamek et al., 2013; Adamek et al., 2018; Boonthai et al., 2018; Kim et al., 2018; Ma et al., 2019). In marine systems, it is possible that secondary or co‐infections with Bacteroidota can confer a threat even in cases with known aetiology and present further challenges for diagnostic, treatment and disease mitigation strategies.

Taken together, it is evident that diseases involving Bacteroidota pathogens in the oceans are complex. Rather than acting exclusively as primary pathogens that follow the ‘one pathogen‐one disease’ paradigm, disease caused by Bacteroidota results from dynamic and multifactorial interactions between the host, pathogen, other biotic agents and the environment. Thus, knowing how and when a host is susceptible to opportunistic pathogens will provide a valuable insight into how diseases caused by Bacteroidota emerge. However, our understanding of opportunistic pathogens is also dependent upon recognizing which commensal Bacteroidota have the capacity to cause disease and the virulence mechanisms they use to inflict damage.

## VIRULENCE MECHANISMS OF PATHOGENIC MARINE BACTEROIDOTA

### Surface attachment and motility

Attachment to host tissue is widely accepted as a prerequisite in the infection process of all pathogens. Marine Bacteroidota typically encode a high number of adhesins in their genomes, and have a general tendency to exist attached to particulate matter in the oceans (DeLong et al., 1993; Fernández‐Gomez et al., 2013; Kirchman et al., 2010), where they can move by surface‐mediated gliding motility. Studies of pathogenic marine Bacteroidota have attributed adhesins as potential virulence traits, including the sea urchin pathogen *Tenacibaculum* sp. F‐2 where attachment to the host, via carbohydrate‐binding receptors, was directly linked to disease outcomes (Taniguchi et al., 2006). The genome of the fish pathogen *T*. *maritimum* was found to encode 17 adhesins, as well as genes for the biosynthesis of exopolysaccharides and carbohydrate‐binding motifs, which were proposed as virulence traits (Pérez‐Pascual et al., 2017a). In addition, surface attachment appears to be regulated by external conditions that are hypothesised to induce changes in surface‐exposed proteins. For example, the host attachment of *Tenacibaculum* sp. F‐2 is positively regulated by temperature, coinciding with the onset of disease occurring in the summer months (Tajima et al., 1997b; Taniguchi et al., 2006). Earlier studies in *T*. *maritimum* also found surface attachment could be enhanced by hydrophobic surface chemistry, bacterial growth stage and nutrient availability (Burchard et al., 1990; Sorongon et al., 1991). These studies collectively signify that the environment can influence the attachment of Bacteroidota to hosts, and further studies should focus on identifying specific adhesion proteins and host receptors that influence attachment under different environmental conditions in order to develop a greater understanding of host–microbe interactions.

Following attachment, Bacteroidota can utilize surface‐associated gliding motility which is mediated by the T9SS. Both the T9SS and gliding apparatus are often implicated in the virulence of pathogenic marine Bacteroidota, especially those characterized via genome sequencing. The role of gliding motility in virulence is clear in pathogens such as *F*. *columnare* and *F*. *psychrophilum*, where mutants deficient in gliding motility‐associated genes lacked adhesion properties, and exhibited reduced biofilm, proteolytic and virulence characteristics (Pérez‐Pascual et al., 2017b; Thunes et al., 2021). However, as gliding motility and the T9SS are common and widespread in Bacteroidota (McBride, 2019; McBride & Zhu, 2013), these functions are unlikely to be inherent virulence traits. Rather, such observations highlight how some Bacteroidota can efficiently utilize common adaptive traits to facilitate pathogenesis.

### Tissue invasion and destruction

Traits centred around the acquisition of iron, polysaccharides and peptides from eukaryotic hosts were strongly associated with virulence phenotypes and often designated as key virulence traits (Table S2). This is not surprising as marine eukaryotes represent a rich source of nutrients for bacteria, and the evolutionary drive for microbes to adopt pathogenic characteristics in order to access these nutrients has long been recognized (Persson et al., 2009; Rohmer et al., 2011). Indeed, the unique nutrient uptake strategies exhibited by Bacteroidota, such as the secretion of CAZyme and peptidase effector proteins via the T9SS, are likely to confer damage to the extracellular matrix of plants and animals, facilitating colonization and access to nutrients. This also correlates with the pathology that is commonly observed, which typically presents as surface lesions, rotting and necrosis (Table 1).

Iron uptake strategies are recognized as general virulence traits in most pathogens and have been identified in multiple pathogens within the phylum Bacteroidota. This includes lobster pathogens *Aquimarina* sp. I32.4 (Ranson et al., 2018) and *Aquimarina* sp. TRL1 (Ooi et al., 2020), and the macroalgal pathogens *Aquimarina* sp. AD1 and *Aquimarina* sp. BL5 (Hudson et al., 2019) which encode multiple iron uptake systems. The iron uptake strategies of *T*. *maritimum* have been studied in the most detail, including the production of multiple siderophores and the ability to use iron from a variety of sources (including transferrin, hemin, haemoglobin and ferric ammonic citrate) (Avendaño‐Herrera et al., 2005; Pérez‐Pascual et al., 2017a).

Some studies also identified pathogens to either encode or produce a repertoire of enzymes that specifically target and degrade host macromolecules. For example, *T*. *maritimum* encodes sphingomyelinase and ceramidase which are predicted to degrade host cell membranes, as well as a range of proteases that can elicit destruction of the host tissue (Nowlan et al., 2020; Pérez‐Pascual et al., 2017a; Rahman et al., 2014). In particular, chondroitin AC lyases produced by *T*. *maritimum* are predicted to function as major virulence factors by targeting the chondroitin sulfate component of fish cartilage and connective tissue (Pérez‐Pascual et al., 2017a; Rahman et al., 2014). This observation was initially supported by studies in the freshwater fish pathogen *F*. *columnare*, where the activity of chondroitin sulfate lyases was correlated with virulence (Stringer‐Roth et al., 2002; Suomalainen et al., 2006). However, the contribution of this enzyme to the virulence phenotype was later attributed, in part, to the competitive advantage it conferred on the pathogen, rather than being an essential virulence trait (Li et al., 2015).

Interestingly, pathogens belonging to the genus *Aquimarina* have a strong tendency to affect hosts rich in complex polysaccharides and encode an enzymatic repertoire orientated towards the carbohydrate makeup of their host. The algal pathogens *Aquimarina* sp. AD1, *Aquimarina* sp. BL5 and *Aquimarina latercula* possess CAZymes, including agarases and carrageenases, which may act on the agar and carrageenan matrix of the algal cell wall (Hudson et al., 2019; Liu et al., 2019; Nedashkovskaya et al., 2005). Likewise, pathogens of crustaceans including *Aquimarina hainanensis* and *Aquimarina* sp. TRL1 exhibited strong chitinolytic activity that potentially degrades the chitin‐rich shell of their crustacean hosts (Chistoserdov et al., 2005; Midorikawa et al., 2020; Ooi et al., 2020; Ranson et al., 2018). However, in other cases, the involvement of host cell wall‐degrading enzymes remains ambiguous, as is the case for the chitinases that are linked to the virulence of the lobster pathogen *Aquimarina* sp. I32.4 despite the destruction of the chitin shell not being apparent in the pathology of epizootic shell disease (Smolowitz et al., 2005). There are also cases of non‐pathogenic Bacteroidota displaying similar phenotypic properties as pathogenic strains. This has been investigated in the algal symbiont *Aquimarina* sp. AD10 which displays a similar agarolytic and carrageenolytic phenotype to the pathogens *Aquimarina* sp. AD1 and *Aquimarina* sp. BL5, yet virulence experiments have demonstrated that it is not pathogenic (Hudson et al., 2019; Kumar et al., 2016).

Thus, while host tissue‐degrading enzymes are associated with virulence phenotypes, their contribution to the disease outcome is complex, and their presence alone is not a determinant of pathogenesis. A reason behind these discrepancies may be due to differences in expression, regulation and catalytic activity of these enzymes that instead determine the virulence potential of Bacteroidota. This has been demonstrated in the plant pathogen *F*. *johnsoniae* where pathogenic isolates were observed to secrete more pectate lyase than non‐pathogenic strains (Liao & Wells, 1986). Although enzyme analysis and mutagenesis studies are limited for marine Bacteroidota, studies that have applied more functional approaches have achieved a deeper insight into the specific factors contributing to virulence. For example, a comparative insight of algal pathogens identified specific enzymes produced by *Aquimarina* sp. AD1 and *Aquimarina* sp. BL5, notably the genes encoding for unique alpha‐agarases (GH96) that were absent in *Aquimarina* sp. AD10 that may instead play a deterministic role in virulence (Hudson et al., 2019). A phenotypic comparison between pathogenic and non‐pathogenic strains of *T*. *maritimum* identified deficiencies in gliding motility in the non‐pathogen that was hypothesised to additionally disrupt the secretion of adhesins and host tissue‐degrading enzymes, rendering it avirulent (Rahman et al., 2014). In addition, gene regulation strategies, such as quorum sensing (QS) have been studied in *T*. *maritimum* and may provide a benefit to pathogens by allowing for the coordinated expression of traits that confer virulence in response to specific environmental conditions (Romero et al., 2010). QS has also been explored in white band disease of coral, where the addition of QS autoinducers correlated with an increase in the abundance of disease‐associated Flavobacteriaceae. Moreover, the addition of a QS inhibitor reduced the abundance of Flavobacteriaceae and inhibited the onset of disease, suggesting a role for QS in the virulence of Flavobacteriaceae pathogens (Certner & Vollmer, 2015; Certner & Vollmer, 2018). However, QS has not been widely studied in Bacteroidota and so its specific contribution to virulence is not fully understood.

### Host evasion and pathogen proliferation

Evading host immune responses is critical for pathogens to establish infection and persist on host tissue. However, only *T*. *maritimum* has been noted for encoding known evasion strategies, including the production of a capsule and a sphingomyelinase haemolytic factor that aids phagosome escape (Nowlan et al., 2020; Pérez‐Pascual et al., 2017a). The genomes of *T*. *maritimum* and some *Aquimarina* pathogens also encode for multiple superoxide dismutase and catalase enzymes, which were proposed as virulence factors since they provide resistance to host oxidative defences (Ooi et al., 2020; Pérez‐Pascual et al., 2017a). Beyond this, host evasion strategies of marine Bacteroidota are not widely reported in the literature. However, as commensal bacteria that normally reside in the host microbiome, Bacteroidota may not need to directly hide from host defences, as they generally appear to cause disease only when the host defences are impaired. Opportunistic Bacteroidota may instead need to encode traits that offer a competitive advantage over other members of the host‐associated microbiome to successfully proliferate and cause disease. This has been suggested for the macroalgal pathogens *Aquimarina* sp. AD1 and *Aquimarina* sp. BL5 which encode for non‐ribosomal peptide synthase (NRPS) and NRPS type 1 polyketide synthase clusters, respectively. These secondary metabolite clusters were found to exhibit homology to genes involved in the synthesis of antimicrobial peptides (Hudson et al., 2019). Likewise, the genome of the lobster pathogen *Aquimarina* sp. I32.4 encodes multiple secondary metabolite clusters (Ranson et al., 2018). Other pathogens also encode numerous antibiotic resistance genes, such as the lobster pathogen *Aquimarina* sp. TRL1, potentially allowing it to resist antimicrobial metabolites produced by other microorganisms (Ooi et al., 2020). These traits may allow Bacteroidota pathogens to resist and suppress bacteria within the microbiota that are beneficial to the host and therefore promote dysbiosis and disease.

**FIGURE 3:**
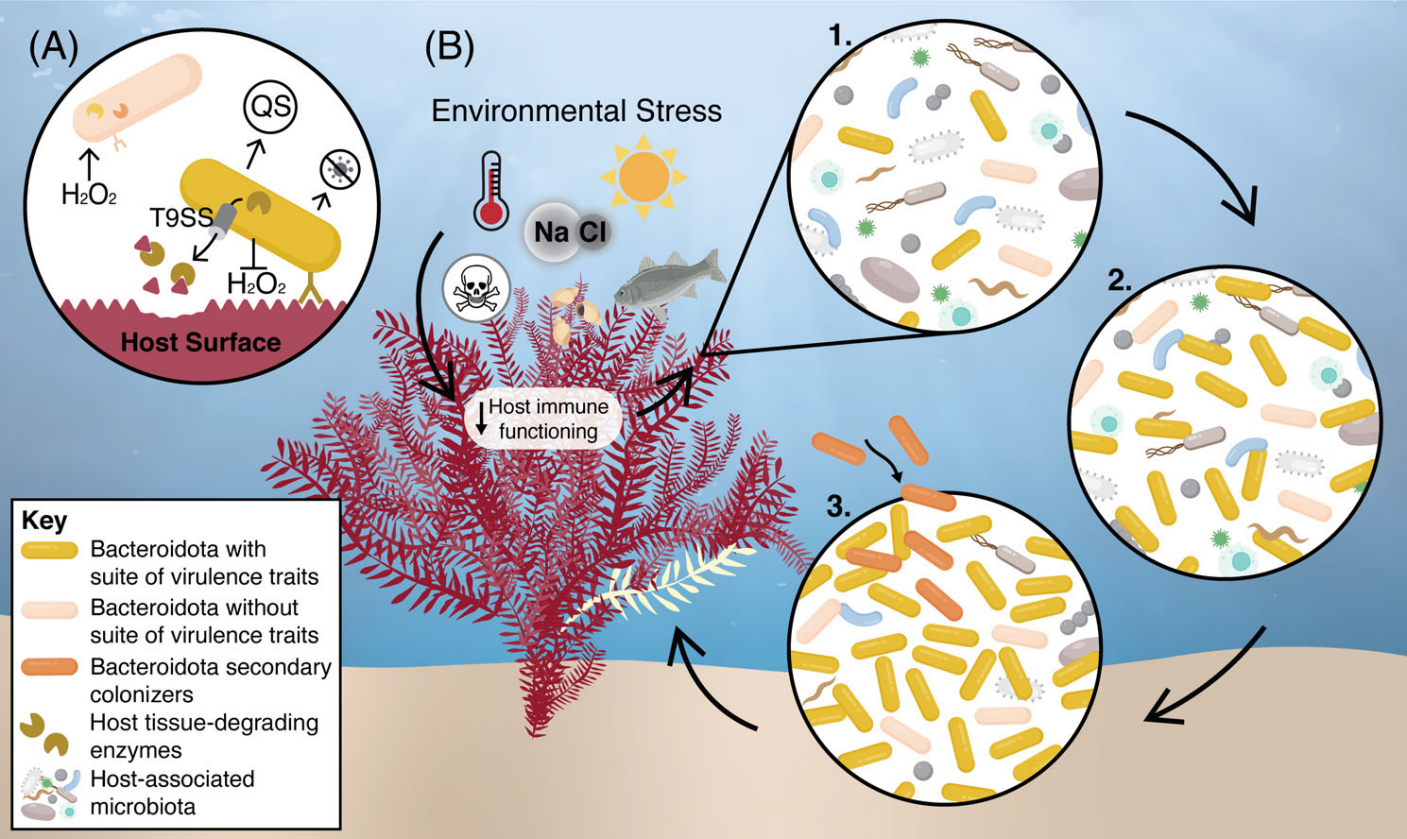
Mechanisms of pathogenesis in marine Bacteroidota. (A) Bacteroidota with the capacity to act as pathogens on marine eukaryotic hosts need to encode an appropriate suite of virulence traits. This may include ‘dual use’ traits (Casadevall et al., 2003) such as CAZymes or peptidases that can specifically target host polymers, the T9SS which can aid in the secretion of these enzymes, adhesins that are specific to host tissue, or the ability to resist oxidative stress. These pathogens may also produce quorum sensing (QS) signals to regulate these traits and antimicrobials to suppress other competitors, which collectively confer a competitive advantage over other microorganisms, including Bacteroidota that do not encode these traits. (B) Using an example of a macroalgal disease, an environmental stress (such as changes in salinity, temperature, light intensity, pollution or the presence of biotic grazers) can weaken host immunity and select for microorganisms that function advantageously under these conditions. Bacteroidota are normally present in the host‐associated microbiota (1) but following the onset of an environmental stress and reduced host immune functioning, Bacteroidota equipped with the necessary virulence traits can rapidly proliferate to degrade and metabolize host macromolecules (2). As infection progresses, secondary Bacteroidota pathogens may colonize and further exploit the available nutrients (3). Tissue damage resulting from enzymatic degradation results in a disease phenotype on the host

Collectively, the virulence of marine Bacteroidota arises from a suite of virulence traits that ultimately confer a competitive advantage allowing for successful colonization and proliferation (Figure 3). Moreover, these traits can generally be considered as ‘dual use’ virulence traits, where they typically function to provide an adaptive advantage, but can also function as virulence traits under certain conditions to cause disease (Casadevall et al., 2003). Utilizing these ‘dual use’ virulence traits may have resulted in many aspects of their pathogenesis being overlooked in studies that aim to characterize the virulence of Bacteroidota pathogens. Therefore, an improved understanding of the role of these traits in virulence may benefit from a greater focus on gene expression, regulation and enzyme activity as well as the effect of environmental conditions on these factors.

## CURRENT LIMITATIONS AND OPPORTUNITIES FOR FUTURE RESEARCH

Despite what has already been inferred through genomics and phenotypic observations, our collective understanding of pathogenicity in marine Bacteroidota is considerably limited. Of the pathogens that have been isolated, few studies have been able to demonstrate their mode of pathogenicity or functionally characterize their virulence traits. This is concerning as an understanding of virulence is critical to our knowledge of disease, predicting outbreaks and improving mitigation, diagnostics, and treatment strategies, particularly in the face of emerging pathogens.

The most striking deficiency in our understanding of virulence in Bacteroidota is the lack of studies analysing gene function, with no studies identified here having produced genetic mutants. This contrasts with studies in Proteobacteria, including marine isolates of *Pseudomonas* and *Vibrio*, where mutagenesis methods are routinely applied, and the function of key genes has been validated and extensively characterized. Although genetic mutants have been successfully created in some non‐marine Bacteroidota, such as *Cytophaga hutchinsonii* (Zhu & McBride, 2014), *F*. *johnsoniae* (Rhodes et al., 2010), *F*. *psychrophilum* (Gomez et al., 2015) and *F*. *columnare* (Li et al., 2015), further work is needed to develop genetic tools that are suitable for marine strains. Thus, whilst greater focus should be given to creating applicable genetic tools, in their absence, studies will need to utilize more phenotypic analysis, enzyme characterization, gene expression and metabolic interaction approaches to allow for a functional understanding of their virulence.

Culture‐independent studies have provided a valuable insight into the role of Bacteroidota, particularly in cases of polymicrobial, dysbiotic diseases or where secondary/saprophytic pathogens are involved by allowing for community‐level changes to be studied. These studies can further benefit from a shift towards metagenomics and transcriptomics to provide a more functionally driven understanding of these complex diseases. Lastly, as environmental conditions appear to greatly influence virulence phenotypes and disease outcomes, studies assessing virulence should include relevant environmental factors in their experimental design.

## CONCLUDING REMARKS

Members of the phylum Bacteroidota thrive in the marine environment owing to their highly competitive nature, unique adaptive traits and ability to form dynamic relationships with eukaryotic hosts. It appears that pathogenic marine Bacteroidota are inherently opportunistic, with virulence arising from a competitive evolutionary drive to exploit host nutrients, often following a perturbation that weakens host immunity (Figure 3). As increasing environmental stressors from climate change, urbanization and pollution put pressure on our marine ecosystems, pathogenic and commensal Bacteroidota equipped with the appropriate suite of virulence traits may therefore have the potential to emerge as a serious threat to ocean life in the future. With the prevalence of disease already increasing in the marine environment, many of which have unresolved aetiology, more investigation is needed to develop a comprehensive understanding of the molecular mechanisms Bacteroidota use to cause disease. With this knowledge, it may be possible to mitigate the emerging threat of marine Bacteroidota, protecting food security and ecologically important marine species alike.

## AUTHOR CONTRIBUTIONS

The study was conceptualized by J.H. and S.E. J.H. wrote the first draft of the manuscript. Both authors contributed to and approved the final version of the manuscript.

## CONFLICT OF INTEREST

The authors declare no conflict of interest.

## Supporting information


**Table S1.** Full list of publications reviewed in this study.
**Table S2**. Reports of Bacteroidetes associated with disease in marine eukaryotic hosts using culture‐dependent methods.

## Data Availability

The full list of publications reviewed in this study is provided in Table S1.
